# The NLRP3 inflammasome: role in the pathobiology of chronic pain

**DOI:** 10.1007/s10787-023-01235-8

**Published:** 2023-04-27

**Authors:** Chen Chen, Maree T. Smith

**Affiliations:** 1 Faculty of Science, School of Chemistry and Molecular Biosciences and School of Biomedical Sciences, Faculty of Medicine, St Lucia Campus, The University of Queensland, Brisbane, Australia; 2 School of Biomedical Sciences, Faculty of Medicine, St Lucia Campus, The University of Queensland, Brisbane, QLD 4072 Australia

**Keywords:** NLRP3 inflammasome, Chronic pain, Inflammatory pain, Neuropathic pain, IL-1β, IL-18

## Abstract

Chronic pain is not only one of the most common health problems, it is often challenging to treat adequately. Chronic pain has a high prevalence globally, affecting approximately 20% of the adult population. Chronic inflammatory pain and neuropathic (nerve) pain conditions are areas of large unmet medical need because analgesic/adjuvant agents recommended for alleviation of these types of chronic pain often lack efficacy and/or they produce dose-limiting side effects. Recent work has implicated the NLRP3 (NOD-, LRR- and pyrin domain-containing protein 3) inflammasome in the pathobiology of chronic pain, especially neuropathic and inflammatory pain conditions. NLRP3 is activated by damage-associated molecular patterns (DAMPs) and pathogen-associated molecular patterns (PAMPs). This in turn leads to recruitment and activation of caspase-1 an enzyme that cleaves the inactive IL-1β and IL-18 precursors to their respective mature pro-inflammatory cytokines (IL-1β and IL-18) for release into the cellular milieu. Caspase-1 also cleaves the pyroptosis-inducing factor, gasdermin D, that leads to oligomerization of its N-terminal fragment to form pores in the host cell membrane. This then results in cellular swelling, lysis and release of cytoplasmic contents in an inflammatory form of cell death, termed pyroptosis. The ultimate outcome may lead to the development of neuropathic pain and/or chronic inflammatory pain. In this review, we address a role for NLRP3 inflammasome activation in the pathogenesis of various chronic pain conditions.

## Introduction

According to the International Association for the Study of Pain (IASP), pain is defined as: “An unpleasant sensory and emotional experience associated with, or resembling that associated with, actual or potential tissue damage” (Raja et al. 2020). Pain can be defined according to its duration (acute or chronic), type (nociceptive, inflammatory, neuropathic) and intensity (mild, moderate, severe). In this review, we have focused on neuropathic pain and chronic inflammatory pain.

### Prevalence

Chronic pain is a condition where pain lasts for more than 3 months and it occurs at least once a week (Debono et al. 2013). It imposes a significant burden on 30% of the world's individuals and economies (Cohen et al. 2021). The prevalence of chronic pain ranges from 11 to 40%, and the cure rate is low (Dahlhamer et al. 2018; Elliott et al. 2002). In a pan-European epidemiological study, the 1-month prevalence of moderate and severe chronic non-cancer pain was 19% (Reid et al. 2011), which was similar to that in Australia (15.0% in males, 16.9% in females (Economics 2019), Denmark (16%) (Harker et al. 2012) and Sweden (18%) (Harker et al. 2012). A study in the United States reported a point prevalence of approximately 20.4% (Dahlhamer et al. 2018). In a systematic review of the burden of chronic pain in the UK, the prevalence of chronic pain was 43.5% and that of moderate-to-severe disabling pain was in the range 10% to 14% (Fayaz et al. 2016). Compared with the young, the elderly have a much higher prevalence of chronic pain at 66% (Reid et al. 2011). Chronic pain is underpinned not only by pathological changes due to disease and neuroplastic changes in the somatosensory nervous system, but also by psychological factors and other influences such as region, social culture, lifestyle and behavior (Edwards et al. 2016; Diatchenko et al. 2013; Mills et al. 2019). Patients suffering from chronic pain have a higher incidence of co-morbid conditions such as depression, anxiety and insomnia (Debono et al. 2013), affecting their ability to work and impairing their quality of life. The annual medical expenses due to chronic pain are high. In Australia, approximately 15.4% of the population suffer from chronic pain, and the annual cost per person is AUD 22,588–42,979 (Economics 2019). From a macro-economic perspective, the direct costs of medical treatments and the associated socioeconomic costs are huge at USD560-635 billion per annum in the USA alone (Institute of Medicine Committee on Advancing Pain Research and Education 2011).

### Unmet medical need

Medications recommended for the pharmacological management of chronic pain depend upon the type of pain that needs to be treated. For example, drugs used to relieve chronic inflammatory pain include nonsteroidal anti-inflammatory drugs (NSAIDs) such as ibuprofen and COX2 inhibitors such as celecoxib (Eccleston et al. 2017). However, treatment with NSAIDs may induce acute hemorrhagic gastritis, peptic ulcer and an increased cardiovascular and renal risk (Shah and Mehta 2012; Enthoven et al. 2016). Additionally, long-term use of COX2 inhibitors is associated with increased risk of cardiovascular side-effects, such as heart attack and stroke (Labianca et al. 2012). First-line drug treatments for the relief of neuropathic pain include tricyclic antidepressants (e.g. amitriptyline), duloxetine and some anticonvulsants (e.g. pregabalin and gabapentin) (Finnerup et al. 2015), but these drugs often lack efficacy and/or have dose-limiting side effects such as orthostatic hypotension, tachycardia, sedation and dizziness (Labianca et al. 2012; Shah and Mehta 2012). Although opioids also relieve pain, they are only recommended as third-line treatments for the relief of neuropathic pain (Vowles et al. 2015). Opioids can be addictive and they evoke a plethora of side effects when used repeatedly (Vowles et al. 2015; Finnerup et al. 2015). Since chronic pain is underpinned by complex mechanisms, pain treatment should ideally be based not only on the severity of pain, but also on its underlying pathogenesis. To address the large unmet medical need for new highly effective and well-tolerated analgesics, many researchers over the past three decades have investigated the pathogenesis of various chronic pain states aimed at identifying novel drug targets for use in novel non-opioid analgesic drug discovery programs. One such target is the NLRP3 inflammasome and so in the following sections, we review a role for the NLRP3 inflammasome in the pathogenesis of neuropathic and chronic inflammatory pain.

### NLRP3 inflammasome structure

**Fig. 1:**
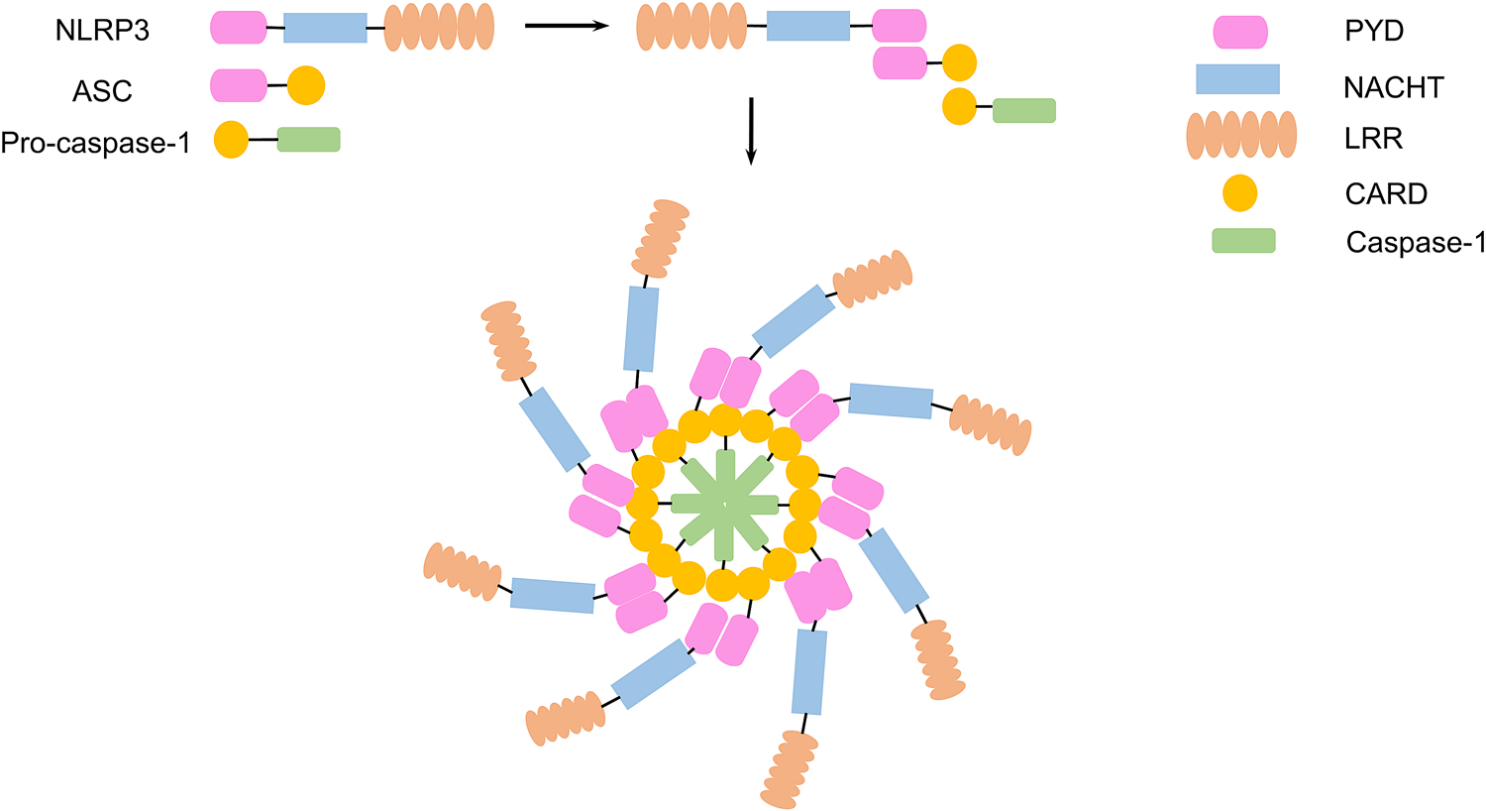
Structure of the NLRP3 inflammasome

The NLRP3 inflammasome is a macromolecular protein complex with a molecular weight in the range 500–700 kDa (Broz and Monack 2011). It is comprised of a sensor (NLRP3), an adaptor (ASC; also known as PYCARD) and an effector (caspase 1) (Bauernfeind and Hornung 2013; Tang et al. 2018; Swanson et al. 2019) (Fig. 1). NLRP3 is a tripartite protein that contains an amino-terminal pyrin domain (PYD), a central NACHT domain and a carboxy-terminal leucine-rich repeat domain (LRR domain) (Swanson et al. 2019; Sutterwala et al. 2014; Tang et al. 2018) (Fig. 1). ASC is an important intracellular junction protein comprising 195 amino acid residues and a molecular weight of 21.5 kDa. It comprises the Pyrin domain (PYD) and the caspase recruitment domain (CARD) (Wen et al. 2011). ASC can connect NLRP3 upstream to Caspase-1 downstream, and interact with PYD to recruit pro-caspase-1 through the CARD domain (Wen et al. 2011). This in turn facilitates NLRP3 to activate the cysteine protease, caspase-1 (Wu et al. 2010). ASC is mainly located in the nucleus of human monocytes/macrophages, but it can be rapidly distributed into the cytoplasm as needed, promoting the activation of the NLRP3 inflammasome (Gris et al. 2010). Caspase-1, also called IL-1 converting enzyme (ICE), is the effector of the NLRP3 inflammasome (Franchi et al. 2009).

## Activation of the NLRP3 inflammasome

**Fig. 2:**
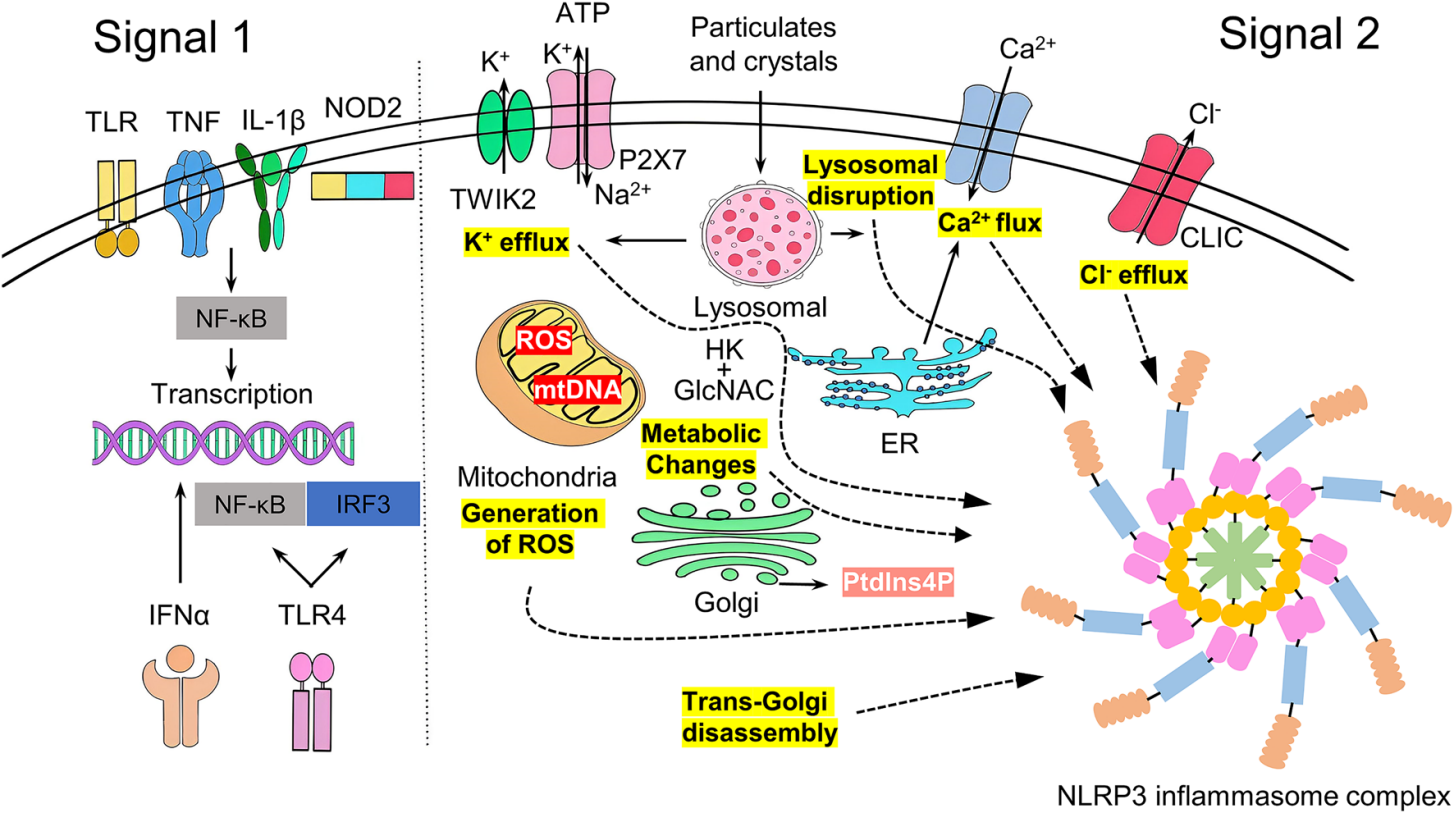
Priming and activation of the NLRP3 inflammasome (TLR: Toll-like receptors; TNF: Tumor necrosis factor; IL-1β: interleukin-1β; NOD2: Nucleotide Binding Oligomerization Domain Containing 2; TWIK2, two-pore domain weak inwardly rectifying K+ channel 2; NF-κB: nuclear factor kappa-light-chain-enhancer of activated B cells; IFN-α: interferon-α; TLR4: toll-like receptors 4; ATP: adenosine triphosphate; IRF3: interferon regulatory factor 3; CLIC: chloride intracellular channel protein; ER: endoplasmic reticulum; P2X7: P2X purinoceptor 7; PtdIns4P: phosphatidylinositol-4-phosphate; ROS: reactive oxygen species; GlcNAc: N-acetylglucosamine; HK: hexokinase)

The NLRP3 inflammasome which is part of the innate immune system, is primed and activated by a broad array of sterile and microbial stimuli (Swanson et al. 2019). Priming (signal 1) upregulates expression of the inflammasome components, NLRP3, caspase 1 and pro-IL-1β (Fig. 2) (Swanson et al. 2019). This activity is induced via recognition of various pathogen-associated molecular patterns (PAMPs) (Franchi et al. 2009) via pattern recognition receptors (PRR) that include toll-like receptors (TLRs) or nucleotide-binding oligomerization domain-containing protein 2 (NOD2) or cytokines such as tumor necrosis factor (TNF) and Il-1β that leads to nuclear factor-κB (NF-κB) activation and gene transcription (Swanson et al. 2019). The 2nd function of priming is stabilization of NLRP3 in an auto-suppressed inactive, but signal-competent state, by induction of post-translational modifications such as phosphorylation, ubiquitylation and sumoylation (Swanson et al. 2019).

Signal 2 (activation) may be provided by numerous PAMPs such as bacteria, viruses and fungi, or damage-associated molecular patterns (DAMPs) including crystals (e.g. urea, cholesterol, silica), particulates (β-amyloid, environmental irritants) and adenosine triphosphate (ATP) (Fig. 2) (Swanson et al. 2019). Once formed, the NLRP3 inflammasome recruits and activates caspase-1 that cleaves pro-IL-1β and pro-IL-18 to produce the corresponding mature cytokines, namely IL-1β and IL18 (Franchi et al. 2009). These cytokines contribute to the removal of the pathogens and activation of the adaptive immune response (Franchi et al. 2009) via induction of cellular stress (Swanson et al. 2019). Precisely how the NLRP3 senses cellular stress and which pathways are induced to culminate in NLRP3 activation and inflammasome formation remain to be fully elucidated (Swanson et al. 2019). Because IL-1β and IL-18 are upstream components of the immune response, they can stimulate the production of a variety of inflammatory mediators, but their excessive production can lead to inflammatory disease (Swanson et al. 2019). Caspase-1 also cleaves the pyroptosis-inducing factor gasdermin D (Wu et al. 2010). The N-terminal fragment of gasdermin D oligomerizes and forms pores in the host cell membrane, leading to cellular swelling, lysis, and release of cytoplasmic contents in an inflammatory form of cell death, called pyroptosis (Wu et al. 2010).

The NLRP3 inflammasome is particularly prominent in inflammatory diseases and it is a Fig. 2 potential target for discovery of novel analgesics as inflammatory mechanisms contribute to the pathophysiology of multiple chronic pain conditions (Tang et al. 2018).

There are multiple mechanisms that may activate the NLRP3 inflammasome. These include potassium (K^+^) or chloride (Cl^−^) efflux, calcium (Ca^2+^) flux, lysosomal disruption, mitochondrial dysfunction, metabolic changes and trans-Golgi disassembly (Swanson et al. 2019).

### K^+^ efflux

Almost all pathways that activate the NLRP3 inflammasome are associated with K^+^ efflux and the NLRP3 inflammasome can be activated in a simple hypokalemic environment (Muñoz-Planillo et al. 2013), which suggests that K^+^ outflow may be a common mechanism (Mathur et al. 2018). NLRP3 inflammasome agonists, such as extracellular ATP, stimulate translocation to the cell surface and activation of purinergic P2X7 receptors which are non-selective channels for Na^+^, K^+^ and Ca^2+^ ions (Franceschini et al. 2015). Interestingly, ATP-induced activation of the P2X7 receptor promoted K^+^ efflux via the K^+^ channel two-pore domain weak inwardly rectifying K^+^ channel 2 (TWIK2) (Di et al. 2018). Additionally, LPS-induced NLRP3 inflammasome activation is dependent upon TWIK2 (Di et al. 2018). Other stimuli that activate the NLRP3 inflammasome by inducing K^+^ efflux include particulates such as alum, silica, sodium urate crystals and calcium pyrophosphate crystals (Muñoz-Planillo et al. 2013). More specifically, K^+^ efflux drives NLRP3 oligomerization (Green et al. 2018).

### Cl^−^ efflux

A role for Cl^−^ efflux in NLRP3 activation is based upon work showing that ATP-induced IL-1β secretion increased or decreased in response to extracellular Cl^−^ concentrations that were low or high respectively, and that chloride intracellular channel proteins (CLICs) are needed for NLRP3 activation by multiple stimuli (Domingo-Fernández et al. 2017; Tang et al. 2017). Translocation of CLICs from the cytosol to the plasma membrane where they form anion channels, is dependent upon the release of mitochondrial reactive oxygen species (mtROS) whereas Cl^−^ efflux occurs downstream of K^+^ efflux (Tang et al. 2017). The role of Cl^−^ efflux is to promote ASC polymerization during NLRP3 inflammasome formation (Green et al. 2018).

### Ca^2+^ flux

A role for Ca^2+^ mobilization in NLRP3 activation is supported by work suggesting that it may occur downstream of both NLRP3 and caspase 1 activation (Katsnelson et al. 2015). However, others showed that K^+^ efflux induced Ca^2+^ flux was transduced by the opening of plasma membrane channels or by the release of endoplasmic reticulum (ER)-linked intracellular Ca^2+^ stores (Di et al. 2018). For example, ATP mobilized Ca^2+^ influx weakly via the P2X7 receptor that was coordinated with K^+^ efflux (Di et al. 2018). This in turn promoted the release of ER-linked Ca^2+^ stores that was followed by the opening of membrane Ca^2+^ channels (Murakami et al. 2012; Yaron et al. 2015).

### Generation of ROS in mitochondria

Release of mtROS and mitochondrial DNA (mtDNA) from dysfunctional mitochondria are key upstream events that induce the assembly and activation of NLRP3 inflammasomes (Jin and Flavell 2010). ROS production induces dissociation of the thioredoxin-interacting protein (TXNIP) from thioredoxin-1(TRX1) in the cytoplasm and promotes its binding to NLRP3, thereby activating the NLRP3 inflammasome (Lane et al. 2013). At the same time, TXNIP may translocate into mitochondria and bind to thioredoxin-2 (TRX2), resulting in mitochondrial dysfunction (Lane et al. 2013). Oxidized mitochondrial DNA can directly activate the NLRP3 inflammasome by acting as a DAMP for NLRP3 activation (Lane et al. 2013; Chen et al. 2017; Zhang et al. 2010). Removal of ROS in macrophages with the ROS scavenger, N-acetylcysteine, reduced intracellular caspase-1 activation that in turn reduced production of the pro-inflammatory cytokine, IL-1β (Zhou et al. 2011). NLRP3 activation can also be inhibited by the nuclear factor erythroid 2-related factor 2 (NRF2) to limit ROS levels (Liu et al. 2017; Su et al. 2021). NRF2 may also attenuate NF-κB activation resulting in downregulation of the expression of multiple NLRP3 components thereby negatively regulating NLRP3 inflammasome activity (Su et al. 2021; Li et al. 2008).

### Lysosomal disruption

Crystalline or granular substances enter cells through macrophage endocytosis resulting in lysosomal acidification and reduced stability of the phagolysosome membrane so that it ruptures and releases the particulates into the cytoplasm, with the net effect being NLRP3 inflammasome activation (Lima et al. 2013; Hornung et al. 2008). Although a role for lysosomal cathepsins in particulate-induced NLRP3 activation was implicated by work using cathepsin inhibitors, this was discounted by findings showing that genetic deletion of individual cathepsins had a minimal effect on NLRP3 activation which in turn suggested that the various cathepsins may have redundant roles in NLRP3 activation (Orlowski et al. 2015). As lysosomal damage in response to particulates activates both K^+^ efflux and Ca^2+^ flux, this implicates a convergence of these processes in many NLRP3 activation pathways (Muñoz-Planillo et al. 2013; Murakami et al. 2012; Katsnelson et al. 2016).

### Metabolic changes

Although the enzyme, hexokinase, is well known to catalyze glucose phosphorylation, it may also have a role in NLRP3 inflammasome activation. For example, in bacterial infection, N-acetylglucosamine released from lysosomes, bound to hexokinase at the mitochondrial surface to induce its relocation to the cytosol (Wolf et al. 2016).This resulted in NLRP3 inflammasome activation independent of K^+^-efflux with mtDNA detected in the cytosol (Wolf et al. 2016). In other work, the NLRP3 inflammasome was activated by free fatty acids (FFAs) derived from dietary sources or by upregulation of FA synthesis (Wen et al. 2011; Moon et al. 2015). Conversely, the anti-inflammatory AMP-activated protein kinase, suppressed FA-induced inflammation by limiting ROS production and activating autophagy which led to inhibition of NLRP3 inflammasome activation (Li et al. 2009b). Metabolic changes such as fasting and caloric restriction also negatively regulate NLRP3 such as that which is mediated by the ketone body, β-hydroxybutyrate, which inhibited NLRP3 activation, suppressed caspase-1 activation and reduced IL-1β release by inhibiting K^+^ efflux (Youm et al. 2015).

### Trans-Golgi disassembly

Trans-Golgi disassembly into vesicles termed the dispersed *trans*-Golgi network (dTGN), may be induced by a range of NLRP3 stimuli (Chen and Chen 2018). Phosphatidylinositol-4-phosphate is a negatively charged phospholipid on the dTGN that recruits NLRP3 through ionic bonding with the conserved polybasic region of the dispersed dTGN, resulting in NLRP3 aggregation, a prerequisite step for downstream ASC oligomerization and caspase-1 activation (Chen and Chen 2018). Observations that K^+^ efflux is essential for NLRP3 recruitment but not dTGN formation, suggest that K^+^ efflux-dependent and mitochondria-dependent NLRP3 activation may be separate pathways that converge on Golgi disassembly, but this requires additional investigation (Swanson et al. 2019).

Apart from the aforementioned pathways that may potentially activate the NLRP3 inflammasome and produce cellular dysfunction and inflammatory diseases, there are multiple mechanisms that may inhibit this process. These latter mechanisms include autophagy (Biasizzo and Kopitar-Jerala 2020), microRNA (miR) post-transcriptional regulation of NLRP3 (Tezcan et al. 2019), and silencing of the heat shock protein family (Zuo et al. 2018; Mi et al. 2021), to negatively regulate NLRP3 inflammasome activation and so provide adequate immune protection and avoid severe tissue damage to the host caused by harmful stimuli.

## Nociceptive (pain) signaling pathway

**Fig. 3:**
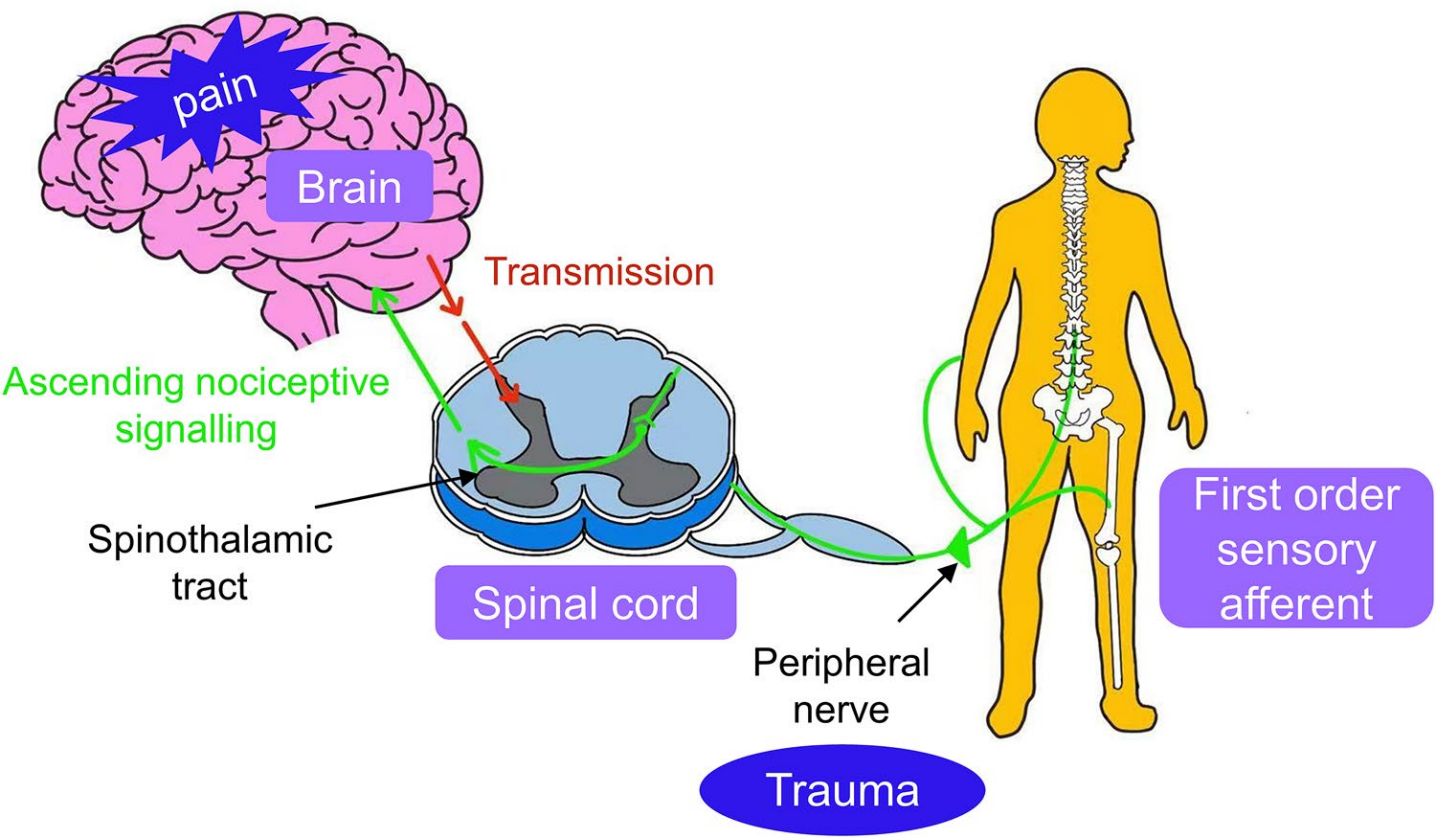
Nociceptive (Pain) signaling pathway

Dysregulation of the NLRP3 inflammasome can lead to excessive production of the pro-inflammatory cytokines, IL-1β and IL-18, leading to severe inflammation and/or a variety of diseases (Jin and Flavell 2010). The pro-inflammatory cytokines, IL-1β and IL-18 are secreted by macrophages and they can interact with their cognate receptors on nerve terminals to sensitize primary afferent sensory nerve fibers and induce pro-nociceptive signaling which is transduced into the dorsal horn of the spinal cord (Chen et al. 2014). This in turn may induce pronociceptive signaling in 2nd order neurons in the spinal cord that is propagated via the spinothalamic tract to higher order structures in the brain where it is interpreted as pain by the cerebral cortex (Doyle et al. 2019) (Fig. 3).

As shown in Fig. 3, key components of the nociceptive signaling (pain) pathway include peripheral nociceptors, first-order primary afferent sensory nerve fibers, the dorsal root ganglia that contain the cell bodies of primary sensory neurons, the dorsal horn of the spinal cord where the central terminals of primary afferent sensory nerve fibers form synapses with second order neurons, the spinothalamic tract and the brain.

## The NLRP3 inflammasome is associated with chronic pain-related diseases

### Neuropathic pain

Neuropathic pain is a major type of chronic pain characterized by spontaneous pain, allodynia and hyperalgesia (Zeilhofer et al. 2012; Bahari and Meftahi 2019). It may be caused by trauma and/or disease resulting in injury to peripheral nerves, posterior roots of the spinal cord, the spinal cord itself, and some central neurons. Neuroinflammatory responses contribute to the development of neuropathic pain following nerve injury with NLRP3 inflammasome activation contributing to these inflammatory responses (Abbaszadeh et al. 2018).

### NLRP3 inflammasome activation in dorsal root ganglia (DRGs) from rodent models of neuropathic pain

#### Central neuropathic pain due to multiple sclerosis

Central neuropathic pain is a major complication of multiple sclerosis (MS) that affects up to 50% of patients with MS (Khan and Smith 2014). This type of pain is generally thought to be transduced by pathological changes in the brain and the dorsal horn of the spinal cord (Khan and Smith 2014). However, recent work in the myelin oligodendrocyte glycoprotein (MOG)-induced experimental autoimmune encephalomyelitis (EAE) mouse model of MS-associated neuropathic pain has implicated an additional component involving activation of the complement system and the NLRP3 inflammasome in the lumbar dorsal root ganglia (DRGs) in the pathobiology of MS-associated neuropathic pain (Yousuf et al. 2019). In particular, there was transient activation of the complement system and prolonged activation of the NLRP3 inflammasome in the lumbar DRGs of EAE-mice that resulted in a small but significant increase in DRG levels of the pro-inflammatory cytokine, IL-1β, and marked hyper-excitability of medium-to-large-diameter Aβ nerve fibers that mediate mechanical hyperalgesia (Yousuf et al. 2019). In other work, chronic oral administration of the NLRP3 inhibitor, MCC950 in a relapsing–remitting EAE mouse model of MS-associated neuropathic pain progressively reversed neuropathic pain behaviour, further implicating a pathobiological role for NLRP3 inflammasome activation in MS-associated neuropathic pain (Khan et al. 2018).

#### Central post-stroke pain

Central post-stroke pain (CPSP) is defined as the neuropathic pain that arises either acutely or in the chronic phase of a cerebrovascular event and is a result of central lesions of the somatosensory nervous system (Liampas et al. 2020). CPSP has a prevalence of 11% with 31% of patients developing neuropathic pain symptoms within one month of stroke onset (Liampas et al. 2020). Ischemia/reperfusion injury (I/R) of the CNS after stroke, leads to cell necrosis or apoptosis, resulting in inflammation and an immune response (Li et al. 2009a). In a mouse model of thalamic hemorrhagic stroke, there was temporal development of pain behavior (mechanical and cold allodynia, heat hyperalgesia) in the hindpaw contralateral to the thalamic lesion, over a 14-day study period post-infarct induction (Huang et al. 2022). Additionally, there was decreased expression of miR-233, a miRNA known to negatively regulate the NLRP3 inflammasome, in the ipsilateral thalamus as early as 1-day post-infarct induction that persisted for the 14-day study duration (Huang et al. 2022). Concurrently, the NLRP3 inflammasome was activated and ipsilateral thalamic expression levels of caspase-1, ASC and NLRP3 were elevated by 1-day post-infarct which persisted until study completion on day 14 (Huang et al. 2022). Together, these data implicate a key role for NLRP3 inflammasome activation in the pathogenesis of post-stroke central neuropathic pain (Huang et al. 2022).

#### Chemotherapy induced neuropathic pain

In other work in a paclitaxel-induced rat model of chemotherapy induced peripheral neuropathy, there was a significant increase in NLRP3 inflammasome expression in CD68-labeled macrophages in the lumbar DRGs which was associated with ectopic firing of primary afferent sensory nerve fibers and the development of neuropathic pain behavior in these animals (Jia et al. 2017).

#### Radiculopathy

In a mouse model of radiculopathy, a neuropathic pain condition notoriously difficult to treat, there was temporal development of mechanical hyperalgesia in the bilateral hindpaws that was fully developed by day 3 and that persisted until at least day 15 of the model (Jin et al. 2017). At day 21 of the model, there was a marked increase in mRNA expression levels of multiple pro-inflammatory mediators including IL-1β, IL-6, TNFα and COX-2 with a fourfold decrease in the mRNA expression levels of the anti-oxidant enzyme, superoxide dismutase, SOD2 (Jin et al. 2017). Treatment of animals with the ‘free radical sponge’ fullerol, alleviated pain behavior and it suppressed otherwise increased mRNA expression levels of TNFα in the lumbar DRGs (Jin et al. 2017). In complementary work using cultured mouse lumbar DRG explants, incubation of explants with TNFα markedly increased the secretion of IL-1β that was suppressed by exposure to fullerol for 24 h (Jin et al. 2017). The increased secretion of IL-1β was underpinned by both increased expression of the NLRP3 inflammasome and caspase-1 and this was inhibited by fullerol (Jin et al. 2017). Together these findings suggest a role for NLRP3 inflammasome activation in the pathophysiology of radiculopathy, a type of neuropathic back and leg pain that is difficult to alleviate.

Lumbar disc herniation (LDH) is an important cause of radiculopathy (Zhang et al. 2017). In a rat model where autologous nucleus pulposus (NP) was implanted into one L5 DRG to simulate LDH, there was increased expression of NLRP3, ASC, Caspase-1, IL-1, IL-18 and other molecules in DRG neurons by one day after surgery and it peaked on day 7 post-implantation (Zhang et al. 2017). In the lumbar DRGs of the same animals (Zhang et al. 2017), there was also upregulated expression of calcitonin gene related peptide (CGRP) which is a pro-nociceptive neuropeptide that is a hallmark neurotransmitter released from small diameter C-fibers (Orita et al. 2013). Treatment of these animals with Bay11-7082, an inhibitor of both NF-kB activation and NLRP3 inflammasome activation, alleviated neuropathic pain behaviour thereby further implicating the NLRP3 inflammasome in the pathobiology of neuropathic pain (Zhang et al. 2017).

#### Neuropathic pain due to partial sciatic nerve injury in mice

In a mouse model of neuropathic pain induced by a partial sciatic nerve ligation (pSNL)-injury, there was a significant decrease in mRNA expression of miR-23a in the lumbar spinal cord (Pan et al. 2018). Conversely, overexpression of miR-23a in the spinal cord prevented pSNL-induced neuropathic pain whereas knockdown of miR-23a induced pain-like behaviour (Pan et al. 2018). In naïve mice, miR-23a knockdown increased spinal cord levels of thioredoxin-interacting protein (TXNIP) which was associated with induction of the NLRP3 inflammasome (Pan et al. 2018). In the spinal cord of pSNL-mice, miR-23a overexpression inhibited the increase of TXNIP and NLRP3 inflammasome activation and alleviated neuropathic pain behavior (Pan et al. 2018). Intrathecal injection of 681-siRNA for 3 consecutive days to knock down TXNIP expression, significantly alleviated pSNL-induced hyperalgesia and mechanical ectopic pain (Pan et al. 2018). Thus, miR-23a was confirmed to regulate neuropathic pain via the NLRP3/TXNIP inflammasome axis in pSNL-mice (Pan et al. 2018).

#### Chronic constriction injury of the sciatic nerve induced neuropathic pain

Many microRNAs (e.g. miR-145, miR-223, miR-23a, miR-183, miR-150) have been implicated in the pathogenesis of neuropathic pain (Ji et al. 2018; Shi et al. 2018; Xie et al. 2017). In work by others using the widely used unilateral chronic constriction injury (CCI) of the sciatic nerve mouse model of neuropathic pain, overexpression of Mir-34c in the lumbar spinal cord alleviated neuropathic pain behavior (Xu et al. 2019). Additionally, there was suppression of the activity of the NLRP3 inflammasome (decreased protein levels of NLRP3, ASC and caspase-1) and decreased inflammatory responses (downregulated production of the pro-inflammatory cytokines, TNFα, IL-1β and IL-18) in the lumbar spinal cord of these animals (Xu et al. 2019).

As noted in Sect. "Generation of ROS in mitochondria", the nuclear factor E2-related factor- 2 (Nrf2) signaling pathway can inhibit activation of the NLRP3 inflammasome by a mechanism involving nuclear translocation of Nrf2 (Liu et al. 2020). This had the same effect as the small molecule NLRP3 inhibitor, MCC950, in terms of alleviating neuropathic pain behavior in the CCI-rat model (Cohen and Mao 2014).

In other work in CCI-mice exhibiting mechanical hypersensitivity in the ipsilateral hindpaws, there was increased expression of connexin-43 hemichannels in lumbar spinal cord astrocytes and there was increased expression of key NLRP3 inflammasome components (NLRP3, ASC, caspase-1) in the lumbar spinal cord of these animals (Tonkin et al. 2018). Hindpaw hypersensitivity was significantly reduced in CCI-mice by intrathecal administration of the connexin-43 mimetic peptide, Peptide5, that blocks hemichannels in spinal cord astrocytes concurrent with a reduction to naïve levels of otherwise increased levels of NLRP3, its adaptor apoptosis-associated spec-like protein (ASC) and caspase-1 protein (Tonkin et al. 2018). Together these findings are consistent with that notion that pain relief evoked by Peptide 5 was transduced by specific inhibition of the NLRP3 inflammasome in the lumbar spinal cord of these mice (Tonkin et al. 2018).

In other work in the CCI-mouse model of peripheral neuropathic pain, intrathecal injection of divanillyl sulfone alleviated mechanical allodynia in the ipsilateral hindpaws (Shao et al. 2021). The mechanism likely involving induction of mitophagy in microglia to promote rapid clearance of reactive oxygen species and attenuate NLRP3 inflammasome activation in the ipsilateral spinal cord (Shao et al. 2021).

#### Painful diabetic neuropathy

Painful diabetic neuropathy (PDN) is a long term microvascular complication of poorly controlled diabetes that is often difficult to alleviate adequately (Fan and Gordon Smith 2022). PDN is the most common form of neuropathic pain globally as it affects up to 50% of patients with diabetes (Bril et al. 2011). Pain symptoms usually present with a "sock" and "glove"-like distribution and no single medication can prevent or completely reverse PDN (Javed et al. 2015). Although the exact pathogenesis of PDN is unclear, there was increased secretion of mRNA encoding NLRP3, ASC, IL-1β, and IL-18 in macrophages and peripheral blood monocytes of patients with type 2 diabetes mellitus (Lee et al. 2013). Additionally, there was increased expression of caspase-1, a key enzyme that causes apoptosis, in these cells from patients with type 2 diabetes (Lee et al. 2013). The secretion of the above related factors is mediated by the NLRP3 inflammasome (Lee et al. 2013). In addition, in a rat model of type 2 diabetes induced PDN, there was increased lumbar spinal cord expression of reactive oxygen species (ROS) as well as protein levels of NLRP3, TXNIP, caspase-1, interleukin 1-beta (IL-1β) and phosphorylated N-methyl-D-aspartic acid receptor subunit 2B (phospho-NR2B) (Wang et al. 2022). In the same rat model, once-daily treatment for 14-days with the ROS scavenger, N-tert-butyl-α-phenylnitrone (PBN) or TXNIP small interfering RNA, alleviated mechanical allodynia and thermal hyperalgesia in the bilateral hindpaws of these animals as well as decreasing protein levels of NLRP3, TXNIP, caspase-1, IL-1β, and phospho-NR2B (Wang et al. 2022). These findings together with complementary data from cultured microglia, implicate a role for ROS signaling through the TXNIP-NLRP3-NR2B pathway in the pathogenesis of PDN (Wang et al. 2022).

In summary, after nerve injury, there is induction of NLRP3 inflammasome activation and upregulated production of proinflammatory cytokines in the lumbar DRGs and in the lumbar spinal cord, which can lead to sensory neuron sensitization and the development of neuropathic pain. Based upon this knowledge, it is clear that new treatments that directly or indirectly inhibit NLRP3 inflammasome activation, have therapeutic potential as novel analgesic agents.

### Inflammatory pain

Inflammatory pain is a common type of chronic pain that may be induced by inflammation associated with tissue damage due to trauma or bacterial infection (Abrahamsen et al. 2008). Inflammasomes regulate inflammation through the lysis of key cytokine precursors resulting in the secretion of mature pro-inflammatory cytokines (IL-1 and IL-18) that contribute to the development and maintenance of chronic inflammatory pain (Schlesinger 2014).

In the Complete Freund’s Adjuvant (CFA)-induced mouse model of chronic inflammatory pain in one hindpaw, there was increased expression of pro-inflammatory markers including NOX4, P-Jak2 / P-Stat3, and NLRP3 in the lumbar spinal cord (Yu et al. 2020). These changes were attenuated in CFA-mice administered muscone, the active ingredient of the Chinese medicine, musk, by intraperitoneal injection once-daily for 7-days (Yu et al. 2020). In addition, pain relief was evoked in the same animals in a dose-dependent manner (Yu et al. 2020), thereby implicating a role for NLRP3 activation in the pathobiology of chronic inflammatory pain.

In other work, intrathecal injection of the highly selective sphingosine-1-phosphate (S1P) receptor 1 subtype (S1PR1) agonist, SEW2871, evoked mechano-allodynia via activation of the NLRP3 inflammasome (increased expression of NLRP3, cleaved caspase 1 and mature IL-1β) in the lumbar spinal dorsal horn of rats (Doyle et al. 2019). These effects in rats were attenuated by treatment with S1PR1 antagonists or with the NLRP3 inflammasome inhibitor, MCC950 (Doyle et al. 2019). Additionally, intrathecal injection of SEW2871 in mice with astrocyte-specific deletions of *S1pr1* did not evoke mechano-allodynia and the activation of cleaved caspase-1 was reduced (Doyle et al. 2019). Together, these findings showed that astrocyte-specific S1PR1 signaling is necessary for SIPR1 agonist-induced NLRP3 activation that underpins SIPR1 agonist induced mechano-allodynia in these animals (Doyle et al. 2019).

### Gout

Gout is a sterile inflammatory disease with hyperuricemia, that is characterized by chronic monosodium urate (MSU) crystal deposition in various tissues (Goldberg et al. 2017). Gouty arthritis is one of the most common inflammatory pain disorders. When the serum urate concentration is higher than or equal to 0.42 mmol/L(7 mg/dL), it is considered clinically to be hyperuricemia (Dalbeth et al. 2021). Hyperuricemia is mainly related to decreased uricase activity (Kratzer et al. 2014), a gene mutation in the uric acid transporter (URAT1) (Tan et al. 2016). Symptoms of gout include joint pain, edema, redness and, in severe cases, disability (Merriman and Dalbeth 2011).

In 2006, Martinon et al. (Martinon et al. 2006) were the first to report that MSU crystals in the joints of patients with gout could activate NLRP3, thereby activating caspase-1 and promoting the maturation of IL-1β and IL-18. MSU crystals are a host-derived DAMP, that activates the NLRP3 inflammasome (Menu and Vince 2011). MSU crystals can cause multiple intracellular changes including mitochondrial injury (Nomura et al. 2015), increased xanthine oxidase (XO) activity (Bauernfeind et al. 2011), ROS production (Zhong et al. 2016), decreased intracellular ATP production (Nomura et al. 2015), inhibition of AMP-dependent protein kinase (AMPK) (Wang et al. 2016) and increased Nrf2 transcription (Jhang et al. 2015). The toll-like receptor (TLR) family of innate immune receptors are transmembrane receptors that bind extracellular ligands to trigger cellular activation and proliferation (Hsu et al. 2019). The innate immune components TLR-2, TLR-4, CD14 (TLR-4 ligand), NLRP3, ASC and caspase-1 are critical for the development of MSU crystal-induced inflammation (Duan et al. 2019; Giamarellos-Bourboulis et al. 2009; Sun and Zhang 2018).

In a mouse model of gouty arthritis involving MSU injection into the ankle joint, this induced ankle oedema, mechanical allodynia, neutrophil infiltration, oxidative stress, NLRP3 inflammasome activation and increased production of the pro-inflammatory cytokines, IL-1β and TNFα (Yin et al. 2020). Treatment of these mice with 4-doses of eucalyptol (anti-inflammatory oil contained in eucalyptus leaves) commencing at 1 h prior to model induction and continuing at intermittent intervals over 2-days, resulted in reduced ankle swelling and attenuation of mechanical allodynia in a manner mirroring mice treated similarly with the nonsteroidal anti-inflammatory drug, indomethacin (Yin et al. 2020). Both eucalyptol and indomethacin significantly reduced the otherwise upregulated mRNA expression of NLRP3, caspase-1, IL-1β and TRPV1 channels in ankle tissue from MSU-mice (Yin et al. 2020). Thus, eucalyptol inhibited MSU-induced activation of the NLRP3 inflammasome in the inflamed ankle joint tissues (Yin et al. 2020). In addition, for MSU-mice treated with the antioxidants, N-acetyl-L-cysteine or 2,2,6,6-tetramethylpiperidine 1-oxyl (Tempol) to reduce ROS generation and oxidative stress, there was a significant decrease in the otherwise upregulated expression of NLRP3, caspase 1, IL-1β and TRPV1 proteins in the ankle joint tissues (Yin et al. 2020). These findings support the notion that the antioxidants reduced NLRP3 inflammasome activation, IL-1β production and TRPV1 over-expression in ankle joint tissues of MSU-mice, in a manner that mimicked the effects of eucalyptol and indomethacin (Yin et al. 2020).β-hydroxybutyric acid, the most abundant ketone in vivo, inhibits activation of the NLRP3 inflammasome by reducing the priming and assembly steps, thereby reducing caspase 1-dependent secretion of IL-1β from neutrophils (Goldberg et al. 2017; Youm et al. 2015). Thus, β-hydroxybutyric acid, is an endogenous anti-inflammatory molecule with potential as a treatment for gout (Goldberg et al. 2017).

In summary, gouty arthritis is a debilitating chronic inflammatory arthritis caused by deposition of MSU crystals in the joints. MSU crystal-induced gouty flares are characterized by IL-1β-driven acute inflammation and intense pain and fever mediated by activation of the NLRP3 inflammasome in neutrophils to activate caspase-1 and increase the release of mature IL-1β and IL-18 in the inflamed joints (Martinon et al. 2006). In patients with gout, chronic deposition and presence of MSU crystals in the joints, facilitates on-going gouty flares underpinned by high systemic levels of NLRP3-derived IL-1β (Goldberg et al. 2017).

### Fibromyalgia

Fibromyalgia (FM) is a clinical syndrome characterized by chronic widespread pain including headaches, pain or cramps in the lower abdomen, fatigue, unrefreshing sleep, cognitive and somatic symptoms and depression (Häuser et al. 2015). In various populations globally, the prevalence of fibromyalgia is in the range 2–4% with the ratio of women to men sufferers at 12:1 (Häuser et al. 2015). The exact pathophysiology of fibromyalgia is unclear but genetic, environmental, psychological and behavioral factors are implicated (Gupta et al. 2007; Kim et al. 2013).

The NLRP3 inflammasome has been implicated in the pathogenesis of FM (Cohen and Mao 2014). In blood mononuclear cells (BMCs) collected from patients with FM, mitochondrial dysfunction was accompanied by increased protein expression of NLRP3, caspase-1 activation and IL-1β expression (Cohen and Mao 2014). In these patients, there was also increased serum concentrations of the pro-inflammatory cytokines, IL-1β and IL-18 and decreased concentrations of co-enzyme Q10 (CoQ_10_) (Cohen and Mao 2014). CoQ_10_ deficiency induced by p-aminobenzoate treatment in blood mononuclear cells (BMCs) in mice, showed that there was NLRP3 inflammasome activation together with marked pain behavior in these mice (Cohen and Mao 2014). In a placebo-controlled clinical trial of oral CoQ_10_ in patients with FM, there was a reduction in NLRP3 inflammasome activation as well as the serum concentrations of IL-1β and IL-18 (Cohen and Mao 2014). Together, these findings implicate a role for NLRP3 activation and CoQ_10_ deficiency in the pathogenesis of FM and suggest that NLRP3 inflammasome inhibition may be a therapeutic opportunity for treating this disease (Cohen and Mao 2014).

In a rat model of reserpine induced fibromyalgia, intraperitoneal administration of the P2X7 purinergic receptor antagonist, Brilliant Blue G (BBG) at 50 mg/kg for 7-days, attenuated pain behavior and it prevented NLRP3 inflammasome activation and consequently inhibited the release of the pro-inflammatory cytokines IL-1β and IL-18 (D'Amico et al. 2021). Together these data suggest that inhibition of the P2X7 receptor to attenuate NLRP3 inflammasome activation may be a potential therapeutic approach for the treatment of fibromyalgia (D'Amico et al. 2021).

### NLRP3 Inhibitors

Although multiple NLRP3 inhibitors have been reported in the past decade (Table 1), most have relatively low potency (uM) for inhibition of NLRP3 and so there is much room for improvement regarding discovery of ligands with nM inhibitory potency. Of the NLRP3 inhibitors listed in Table 1, only dapansutrile (OLT1177) has entered clinical trials in patients with chronic pain (gout) to date.

**Table 1:** Characteristics of NLRP3 inhibitors

No	Inhibitor	~ IC_50_	Inhibition mechanism	Specificity	Clinical status	References
1	MCC950 (CP-456773)	8 nM	Binds Walker B motif; NACHT ATPase inhibitor	NLRP3	Phase II in patients with cryopyrin associated periodic syndrome (CAPS)	(Coll et al. 2015, 2019; Swanson et al. 2019)
2	CY-09	5 µM	Binds Walker A motif; NACHT ATPase inhibitor	NLRP3	–	(Erdag et al. 2023; Swanson et al. 2019)
3	Oridonin	0.5 µM	Binds irreversibly to NLRP3 Cys279 and blocks NLRP3-Nek7 interaction	NLRP3	-	(He et al. 2018; Swanson et al. 2019)
4	Tranilast	25–50 µM	Binds NACHT and inhibits the NLRP3–NLRP3 interaction	NLRP3	Approved	(Swanson et al. 2019; Huang et al. 2018)
5	MNS	2 µM	NACHT ATPase inhibitor	NLRP3	-	(Swanson et al. 2019; El-Sharkawy et al. 2020)
6	OLT1177 dapansutrile	1–100 nM (mouse) 1 µM (human)	NACHT ATPase inhibitor	NLRP3	Phase II in patients with gout	(Swanson et al. 2019; Marchetti et al. 2018)
7	Bay11-7082	5 µM	NACHT ATPase inhibitor	NLRP3 NLRC4	–	(Swanson et al. 2019; Juliana et al. 2010)
8	BOT-4-one	0.59–1.28 µM	Alkylation; NACHT ATPase inhibitor	NLRP3 NLRC4	–	(Swanson et al. 2019; Shim et al. 2017)
9	Parthenolide	5 µM	NACHT ATPase inhibitor and caspase 1 inhibitor	NLRP3 NLRC4 AIM2 NLRP1	–	(Swanson et al. 2019; Zahid et al. 2019)
10	INF39	10 µM	NACHT ATPase inhibitor	NLRP3	–	(Swanson et al. 2019; Shi et al. 2021)
11	Ginsenoside Rg3	12.75 µM	Inhibition of the NEK7-NLRP3 interaction	NLRP3 NLRC4 AIM2	Approved	(Shi et al. 2020)
12	YQ128	0.3 µM	NACHT ATPase inhibitor	NLRP3	–	(Jiang et al. 2019)
13	Licochalcone B	2.16 µM	Inhibition of the NEK7-NLRP3 interaction	NLRP3	–	(Cao et al. 2020)
14	RRx-001 bromonitrozidine	116.9 nM	Inhibition of the NEK7-NLRP3 interaction	NLRP3	Phase III in patients with small cell lung cancer (SCLC)	(Jayabalan et al. 2023)

## Conclusion

The NLRP3 inflammasome is a key component of the innate immune system that plays an essential role in the pathophysiology of various chronic inflammatory pain conditions as well as multiple types of central and peripheral neuropathic pain. Hence, targeting of the NLRP3 inflammasome may be an effective approach to address the large unmet medical need for a new generation of well-tolerated, safe and highly effective analgesic agents for the relief of chronic inflammatory pain and for alleviating neuropathic pain.

## Data Availability

Not applicable.
